# The Use of Angle-Independent M-Mode in the Evaluation of Diaphragmatic Excursion: Towards Improved Accuracy

**DOI:** 10.7759/cureus.17284

**Published:** 2021-08-18

**Authors:** Jessica Schleifer, Hamid Shokoohi, Lauren Ann J Selame, Andrew Liteplo, Sigmund Kharasch

**Affiliations:** 1 Department of Anesthesia and Intensive Care Medicine, University Hospital Bonn, Bonn, DEU; 2 Department of Emergency Medicine, Massachusetts General Hospital, Boston, USA; 3 Department of Emergency, Brigham and Women's Hospital, Boston, USA

**Keywords:** diaphragmatic excursion, ultrasound, diaphragm, critical care, angle independent m-mode

## Abstract

Assessment of diaphragmatic function has been well described in the intensive care setting as well as in emergency medicine and pediatrics. Conventional M-mode evaluation of diaphragmatic excursion is frequently associated with over and under-estimations of diaphragmatic excursion. Angle-independent M-mode allows free rotation and movement of the analysis line to obtain M-mode images in a direction that more accurately reflects diaphragmatic excursion. In order to provide a standardized approach to the evaluation of diaphragmatic excursion with angle-independent M-mode, we propose a landmark-based approach utilizing the spine in order to target the same diaphragmatic segment consistently throughout the diaphragmatic analysis. While the proposed approach is not intended to replace current methods, it may improve accuracy and inter-rater reliability. The relevant background, as well as three patient cases, are presented demonstrating the use of a landmark-based approach in the emergency department. Angle-independent M-mode may provide a more accurate and consistent evaluation of diaphragmatic excursion, an examination that can be used to guide clinical care and anticipate outcomes.

## Introduction

Assessment of diaphragmatic function has been well described in the intensive care setting as well as in emergency medicine and pediatrics. The use of ultrasound to evaluate diaphragmatic motion was first described in the 1970s [1,2]. More recently, assessment of diaphragmatic function with ultrasound has been described in neuromuscular disease [3], ventilator-induced diaphragmatic dysfunction [4-7], bronchiolitis [8], cardiothoracic surgery [9], endotracheal tube placement [10], interstitial lung disease [11], and diaphragmatic strain using speckle tracking [12]. The diaphragm is the principal muscle of respiration and consideration of diaphragmatic fatigue and dysfunction as potential etiologies of respiratory distress may enhance patient evaluation and guide treatment, especially in cases of unexplained dyspnea [13]. Ultrasound evaluation of the diaphragm offers a real-time dynamic evaluation of this important respiratory muscle by the bedside clinician, something he/she would not be able to accomplish in the emergency setting without ultrasound.

Conventional utilization of M-mode to evaluate diaphragmatic function includes assessment of diaphragmatic thickness at the zone of apposition [4,5] and diaphragmatic excursion [4-6,14]. The domain of diaphragmatic excursion is usually measured using a subcostal window either between the midclavicular and anterior axillary line [8] or the posterior axillary approach with a coronal view [5,6]. In the following cases, an ultrasound landmark-based approach is proposed. In this approach, the probe marker is oriented cephalad with the transducer placed in the midaxillary line, similar to that used in the Focused Assessment with Sonography in Trauma examination. The operator should visualize the ipsilateral hemidiaphragm and vertebral spine. The use of angle-independent M-mode in this approach may lead to a more accurate measurement of diaphragmatic excursion.

The current conventional use of M-mode does not ensure alignment of the M-mode cursor with the true axis of diaphragmatic excursion and there is concern that this may lead to measurement inaccuracies. Angle-independent M-mode (also known as anatomic or post-processing M-mode) allows free rotation and movement of the analysis line to obtain M-mode images in any direction. Comparison of conventional M-mode and angle-independent M-mode in clinical practice has shown that angle-independent M-mode may in fact more accurately reflect diaphragmatic excursion and assessment of diaphragmatic dysfunction. In two small, non-randomized trials, traditional M-mode measurements overestimated diaphragmatic excursion compared to angle-independent M-mode [15,16].

Although the excursion measurement is sensitive to both angle orientation and spatial plane on anatomical orientation, on the average image, the effect of angle errors can be minimized by consistent use of angle-independent M-mode. Through the following series of cases, we propose a standard approach for diaphragm excursion measurements combining angle-independent M-mode with ultrasound landmark guidance using the spinal vertebrae in order to standardize the spatial plane and target the same diaphragmatic segment consistently throughout the diaphragmatic assessment. While the proposed approach is not intended to replace current methods, it may improve accuracy and inter-rater reliability.

## Case presentation

Technique

**Figure 1:**
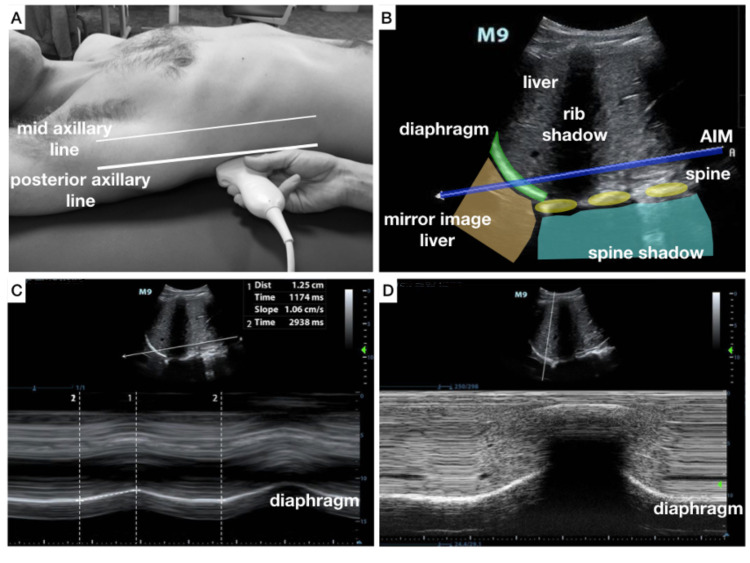
A demonstration of the steps to obtain landmark-based probe orientation and perform angle-independent M-mode to measure diaphragmatic excursion A: Probe placement for visualization of the diaphragm at the posterior-axillary line for a coronal view of the diaphragm. B: B-mode image obtained in coronal view, angle-independent M-mode with optimal probe orientation. C: Activation of angle-independent M-mode with cursor parallel to the spine and demonstrating diaphragmatic excursion measurements. D: Comparison with conventional M-mode on the same patient depicts difficulty in visualization of full diaphragm excursion due to artifacts.

We propose using the vertebral spine as a reference standard to direct optimal spatial orientation, and this can be reliably attained through landmark-based ultrasound guidance (Figure 1).

**Figure 2:**
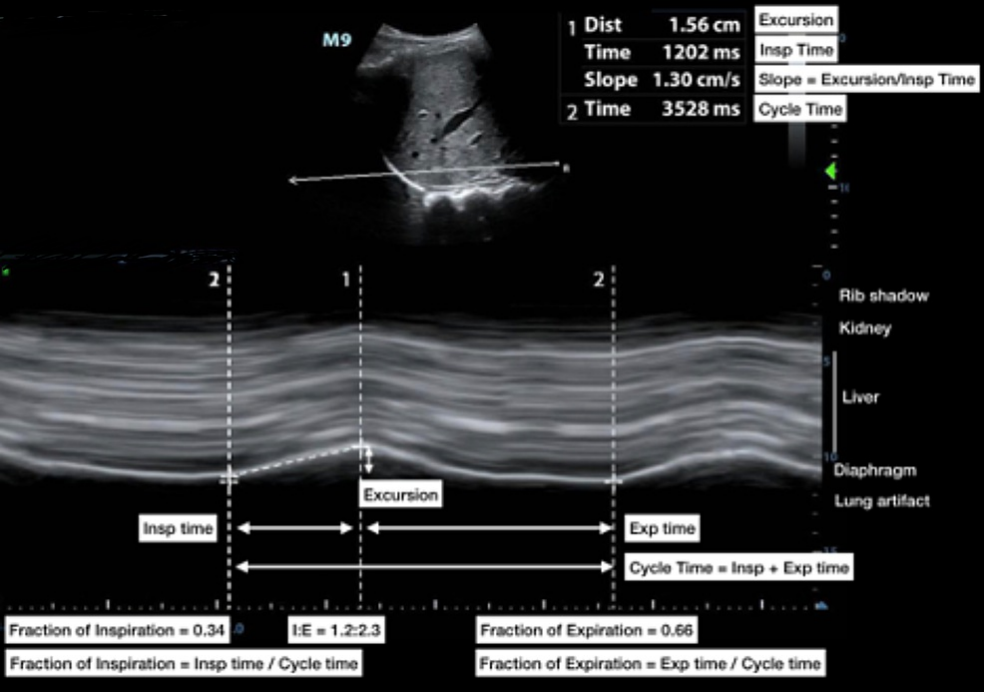
Annotated depiction of diaphragmatic excursion calculation in a healthy adult Right hemidiaphragm excursion parallel to the spine in a healthy 22-year-old female including potential measurements. Excursion during quiet respiration. Insp = inspiration; Exp = expiration; I:E = inspiratory to expiratory time ratio. Fraction of inspiration = Insp time/cycle time = 1202 ms/3528 ms = 0.34; fraction of expiration = Exp time/cycle time = (3528 ms-1202 ms)/3528 ms = 0.66; I:E ratio = 1; Exp time/Insp time = 1: 2326 ms/1202 ms = 1:1.9; inspiratory slope = excursion/Insp time = 1.56 cm/1202 ms = 1.3 cm/s.

Aligning the image orientation and M-mode cursor parallel to the spine within the direction of diaphragmatic motion allows for a standardized assessment of the diaphragm, which facilitates quantitative analyses of diaphragmatic function. To illustrate this technique, we used the Mindray M9 ( Mindray, Shenzhen, China) with either the curvilinear or phased array probe placed in the posterior-axillary line with the probe marker cephalad to obtain a coronal view. The ultrasound image obtained demonstrates the spine in the far-field as a hyperechoic scalloped line with posterior shadowing. The diaphragm is seen cephalad to the spine on-screen left as it arcs towards the probe. With respect to the diaphragm, the abdominal organs (liver, spleen, and kidney) are visualized caudal and lung artifacts cephalad. The motion of the diaphragm during respiration can be observed most clearly at one-quarter of the distance from the spine to the rib cage. The angle-independent M-mode is activated and turned parallel to the spine at the appropriate distance to analyze the diaphragmatic motion. In this position, lung artifacts are less prominent as compared to conventional M-mode. All measurements can be performed with this technique including diaphragmatic excursion, slope, inspiratory and expiratory times, index of inspiration/expiration-time, and respiratory rate (Figure 2).

Case 1: A 13-year-old patient with an asthma exacerbation

A 13-year-old male with a history of mild intermittent asthma presented with two days of shortness of breath, which worsened overnight. It was associated with a frequent cough and a “tight chest.” He received three albuterol nebulizers and was brought to the emergency department (ED). In the ED, he had a pulse of 114 beats per minute, blood pressure of 97/53 mmHg, respiratory rate of 28 breaths/minute, and an oxygen saturation of 99% on room air. On physical exam, he was noted to be in mild respiratory distress with bilateral wheezing. He was treated with three ipratropium/albuterol nebulizers and one dose of oral dexamethasone. He was discharged four hours later.

**Figure 3:**
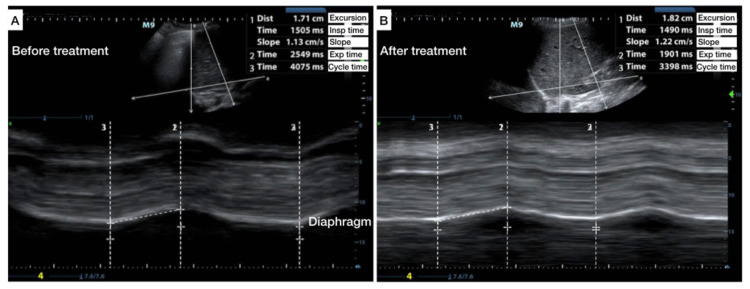
Diaphragmatic excursion in an asthmatic child Diaphragmatic assessment of the right hemidiaphragm using angle-independent M-mode before and after medical treatment of an asthmatic child.

An ultrasound was performed at the time of admission and discharge. Both exams were negative for B-lines, consolidations, or pleural effusions. The left and right hemidiaphragm demonstrated equal pre-treatment excursion. The diaphragmatic excursion increased by 6% after treatment. The total time of expiration shortened from 2549 ms to 1901 ms, the I:E ratio improved from 1:1.7 to 1:1.3, and the slope increased from 1.1 cm/s to 1.2 cm/s (Figure 3).

Case 2: A 73-year-old ventilator-dependent patient with weaning failure and hypoxia

A 73-year-old ventilator-dependent male was transferred to the ED from a rehabilitation facility with worsening oxygenation, leukocytosis, and altered mental status. The patient was ventilator-dependent with a tracheotomy for the past two months due to end-stage fibrotic lung disease. Weaning from ventilation had not been successful and he acutely desaturated overnight. The patient was started empirically on vancomycin and meropenem and transferred to the ED. In the ED, he had a pulse of 123 beats per minute, a blood pressure of 121/63 mmHg, a respiratory rate of 33 breaths/minute, and an oxygen saturation of 88% on mechanical ventilation. Ventilator settings were assist control/volume control, fraction of inspired oxygen (FiO2) 100%, and positive end-expiratory pressure of 8 mmHg with resultant peak inspiratory pressure of 38 mmHg. Cardio-pulmonary exam revealed rales at the lower lung bases without wheezing or rhonchi, tachycardia, and normal S1 and S2 without murmurs. The remainder of his physical exam was unremarkable. A chest x-ray revealed diffuse bilateral interstitial lung opacities consistent with known chronic interstitial changes and bibasilar opacities concerning for multifocal pneumonia.

**Figure 4:**
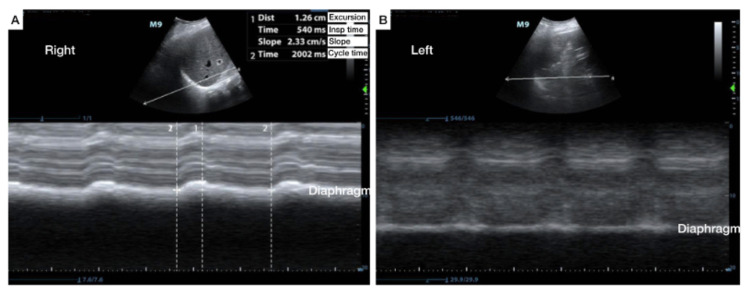
Diaphragmatic excursion in an adult with pneumonia and diaphragmatic paralysis Comparing right and left hemidiaphragmatic excursion in a ventilated patient with end-stage fibrotic lung disease and pneumonia using angle-independent M-mode. Limited diaphragmatic movement on the affected side is suggestive of paralysis.

A lung ultrasound demonstrated small subpleural consolidations, B-Lines bilaterally, and a left base consolidation with static air bronchograms. Diaphragmatic ultrasound revealed a diaphragmatic excursion of the right hemidiaphragm of 1.3 cm, with a slope of 2.3 cm/s, an inspiratory time of 540 ms, and a cycle time of 2002 ms during ventilation. The left side, however, showed no excursion, indicating left diaphragmatic paralysis (Figure 4). The patient was admitted to the intensive care unit for continued treatment of his pneumonia.

Case 3: An 82-year-old patient with unexplained dyspnea

An 82-year-old female presented to the ED with sudden onset of shortness of breath associated with diaphoresis, nausea, and anxiety. The patient reported she felt hot and fatigued in the afternoon when she experienced sudden onset of shortness of breath. Per Emergency Medical Services, her room air oxygen saturation was 90% with improvement on 2 L of oxygen by nasal cannula upon arrival. Her past medical history was remarkable for hypertension, chronic obstructive pulmonary disease, diabetes, chronic renal disease, congestive heart failure, and anxiety. In the ED, she had a pulse of 60 beats per minute, a blood pressure of 143/69 mmHg, a respiratory rate of 22 breaths/minute, and an oxygen saturation of 93% on room air. She had a normal cardiac exam, mild pitting edema of both legs, and strong peripheral pulses. On lung auscultation, she had no crackles or wheezing, but she had absent breath sounds in the right lower chest. A chest X-ray demonstrated elevation of the right hemidiaphragm without signs of pulmonary edema, pneumonia, pleural effusion, or pneumothorax.

**Figure 5:**
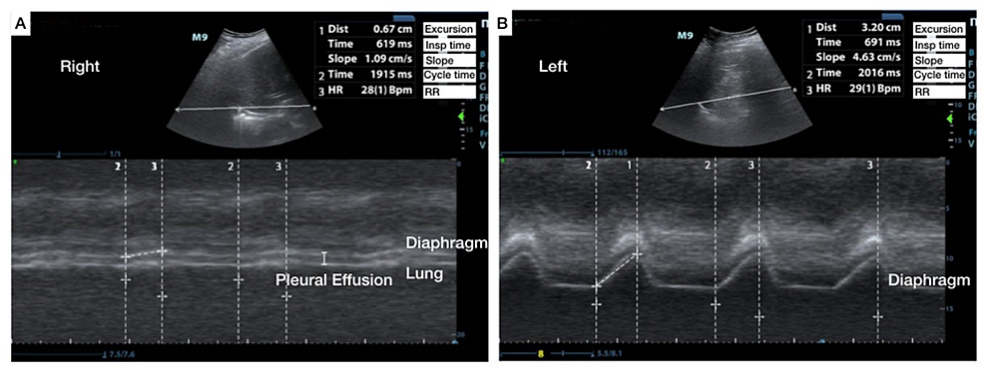
Demonstration of impaired diaphragmatic excursion and a pleural effusion Comparing right and left hemidiaphragm excursion in a patient with right hemidiaphragm paralysis and respiratory distress.

Ultrasound of the lung showed a small right-sided pleural effusion. Ultrasound of diaphragm showed right hemidiaphragmatic excursion was 0.7 cm and slope was 1.1 cm/s. The left hemidiaphragm excursion was notable for compensatory diaphragmatic excursion of 3.2 cm and a slope of 4.6 cm/s. The respiratory rate was 28/min consistent with respiratory distress. Overall, diaphragmatic ultrasound demonstrated severe impairment of right hemidiaphragmatic function, the likely culprit for her sudden-onset respiratory distress (Figure 5).

## Discussion

Diaphragm ultrasound has been more recently proposed as a simple, non-invasive, dynamic bedside quantification of diaphragm function, but typically its use is operator dependent and limited by patient compliance and habitus [17,18]. The use of point-of-care ultrasound for the assessment of diaphragm function has infrequently been described in the emergency medicine literature. Limited case reports of diaphragmatic rupture detected during the performance of the Focused Assessment with Sonography in Trauma exam have been reported in blunt trauma patients. Findings have included diminished diaphragm excursion upon visual inspection and diaphragmatic paralysis on M-mode [19,20]. In a study of 61 infants presenting to an ED with bronchiolitis, diminished diaphragm thickening fraction and a higher inspiratory slope were predictive of the future need for respiratory support during hospital admission [8]. Diaphragmatic ultrasound has been compared to chest radiography to determine endotracheal tube placement in a study of 127 intubated patients in a pediatric emergency room but was found to be inferior to chest radiography for endotracheal tube placement [10].

We illustrated diaphragm ultrasound in three patients with respiratory distress by using a standard ultrasound landmark approach, which utilizes the spine in order to precisely target the same diaphragmatic segment in the evaluation of diaphragmatic excursion with angle-independent M-mode. Angle-independent M-mode allows for fewer orientation errors compared to conventional M-mode and the use of the spine as a reference landmark in angle-independent M-mode may improve the reproducibility of diaphragmatic assessment, comparison with the contralateral hemidiaphragm, and quantitative analysis of dynamic diaphragmatic excursion. The simplicity of use of a landmark already used in lung ultrasound, the spine, may allow for more rapid performance of examination as performers are likely comfortable with spine identification, which is already used in evaluation for pleural effusions, hemothorax, and pneumonia. Use of a standard ultrasound landmark (vertebral spine) approach rather than cutaneous approaches (subcostal, posterior axillary line, etc.) to diaphragmatic evaluation may lead to improved precision and accuracy through examination reproducibility and simplicity. This may ultimately benefit patients with acute respiratory complaints and chronic disease requiring interventions guided by diaphragmatic function.

Potential limitations of an angle-independent M-mode approach to diaphragm evaluation include obese patients who may have more difficulty visualizing spines by ultrasound. Through the use of the posterior axillary line approach bowel gas should not obscure identification of the spine and diaphragm; however, bowel gas may limit evaluation when using the subcostal approach. The angle-independent M-mode function, while available on a number of portable ultrasound machines (Mindray M9 and ME8, Siemens Acuson X600, GE Venue), may not be available on all machines thereby limiting the use of this application.

## Conclusions

The use of ultrasound in the assessment of diaphragmatic function can provide clinicians with valuable information with regard to pulmonary function in the emergency department and critical care settings. Compared to traditional M-mode, angle-independent M-mode may more accurately reflect diaphragmatic excursion and assessment of diaphragmatic dysfunction. A standardized, landmark-based approach utilizing the spine as a reference and angle-independent M-mode is proposed and may provide improved, consistent, and objective quantitative assessment of diaphragm excursion. Future studies comparing this approach using ultrasound landmark guidance to other assessments of diaphragmatic function should be performed.
